# Adherence to national trauma triage criteria in Norway: a cross-sectional study

**DOI:** 10.1186/s13049-024-01306-x

**Published:** 2024-12-18

**Authors:** Einar Frigstad Hoås, Waleed Mohammed Majeed, Olav Røise, Oddvar Uleberg

**Affiliations:** 1 Institute of Clinical Medicine, Faculty of Medicine, University of Oslo, Oslo, Norway; 2 Norwegian Trauma Registry, Division of Orthopedic Surgery, Oslo University Hospital, Oslo, Norway; 3 Department of Emergency Medicine and Pre-Hospital Services, St. Olav’s University Hospital, 7006 Trondheim, Norway; 4 The Norwegian Air Ambulance Foundation, Oslo, Norway; 5 Department of Circulation and Medical Imaging, Norwegian University of Science and Technology, Trondheim, Norway

**Keywords:** Trauma, Wounds and injuries, Injuries, Triage

## Abstract

**Background:**

Norwegian hospitals employed individual trauma triage criteria until 2015 when nationwide criteria were implemented. There is a lack of empirical evidence regarding adherence to Norwegian national criteria for activation of the trauma team (NTrC) and the decision-making processes regarding trauma team activation (TTA) within Norwegian trauma hospitals. The objectives of this study were to investigate institutional adherence to the NTrC and to investigate similarities and differences in the decision-making process leading to TTA in Norwegian trauma hospitals.

**Methods:**

A digital semi-structured questionnaire regarding adherence to criteria, TTA decision-making and criteria documentation was distributed to all Norwegian trauma hospitals (n = 38) in the spring of 2022. Contact details of trauma coordinators and registrars were provided by the Norwegian Trauma Registry secretariat. Follow-up telephone interviews were conducted at the investigator’s discretion in cases of non-respondents or need to clarify answers.

**Results:**

Thirty-eight trauma hospitals were invited to answer the survey, where 35 hospitals responded (92%), making 35 the denominator of the results. Thirty-four (97.1%) hospitals stated that they followed NTrC. Thirty-three (94.3%) of the responding hospitals provided documentation of their criteria in use, of which twenty-eight (80%) of responding hospitals adhered to the NTrC. Three (8.6%) hospitals employed a tiered TTA approach with different sized teams. In addition four hospitals (11.4%) used specialized teams to meet the needs of defined patient groups (e.g. geriatric patients, traumatic brain injury). Twenty-one (60%) of the responding hospitals had written guidelines on who could perform TTA and in 18 hospitals (51.4%) TTA could be performed by pre-hospital personnel. Twenty-three (65.7%) of the hospitals documented which criteria that were used for TTA.

**Conclusion:**

There is good adherence to the national criteria for activation of the trauma team among Norwegian trauma hospitals after implementation of national guidelines. Individual hospitals argue the use of certain local criteria and trauma team activation decision-making processes to increase their precision in specific patient populations and demographics. Further steps should be done to reduce the variation in TTA decision-making processes among hospitals and improve documentation quality.

**Supplementary Information:**

The online version contains supplementary material available at 10.1186/s13049-024-01306-x.

## Background

An important part of the trauma system is to rapidly identify, diagnose and treat potentially life-threatening injuries. To achieve this, field triage guidelines, commonly referred to as trauma triage criteria, were introduced in 1976 with several later revisions [1]. These criteria consist of specific variables relating to physiologic deviations, anatomical injuries, specific mechanism of injury (MOI) and special circumstances (e.g. age, co-morbidity, medication use) and is an essential tool in the early detection of severe injury [2]. Originally, in the US context, these criteria were designed to triage patients to trauma centers if they fulfilled the criteria [2]. As more structured trauma systems were developed in Norway and other Scandinavian hospitals at the beginning of the early 2000s, the same criteria were adopted more or less unadjusted as trauma team activation criteria [3].

These criteria should ideally have both a 100% sensitivity (identify all severe injuries, i.e. no undertriage) and a 100% specificity (no TTA in minor injuries, i.e. no overtriage) [4]. Undertriage is commonly defined as patients with severe injuries (i.e., Injury Severity Score [ISS] > 15), but who do not receive TTA. Conversely, patients who are not severely injured (i.e., ISS < 15), but are identified as severely injured and treated as such are commonly referred to as overtriage [4, 5]. However, there is a challenge presented in attempting to balance overtriage and undertriage. The American College of Surgeons—Committee on Trauma (ACS-COT) have suggested quality indicators to keep rates of overtriage ≤ 35% and undertriage ≤ 5% to avoid increased mortality [6]. A recent systematic review across all ages found undertriage rates from 14 to 34% and overtriage rates from 12 to 31% [7]. Several Norwegian studies have found substantial overtriage rates ranging from 55 to 91.5% [4, 8–11]. These observations indicate that there are challenges to the current system.

In 2010, a study by Larsen et al. found substantial differences in TTA criteria among Norwegian hospitals. The study described 156 different variables used, where no single criterion was common to all hospitals and a wide variation of physiological threshold values existed [3]. In 2015 Norwegian national criteria for activation of the trauma team (NTrC) were implemented following the release of recommendations on how to organize a trauma system aiming to optimize trauma care in a Norwegian context [12]. The objectives of this study were to investigate institutional adherence to the NTrC and to investigate similarities and differences in the decision-making process leading to TTA in Norwegian trauma hospitals.

## Methods

### Study design

The study was a cross-sectional study involving all Norwegian hospitals classified in the Norwegian trauma systems framework as a trauma hospital [12]. The study followed the ‘Strengthening the reporting of observational studies in epidemiology’ (STROBE) recommendations for reporting of observational cohort studies [13].

### Setting

Norway is a long and narrow Scandinavian country with a population of 5.4 million people [14]. Much of the country is uninhabited with 82% of the population living in urban areas [15]. The Norwegian health system is publicly funded, and a fundamental principle is to ensure equal care quality independent of the residential pattern.

**Fig. 1:**
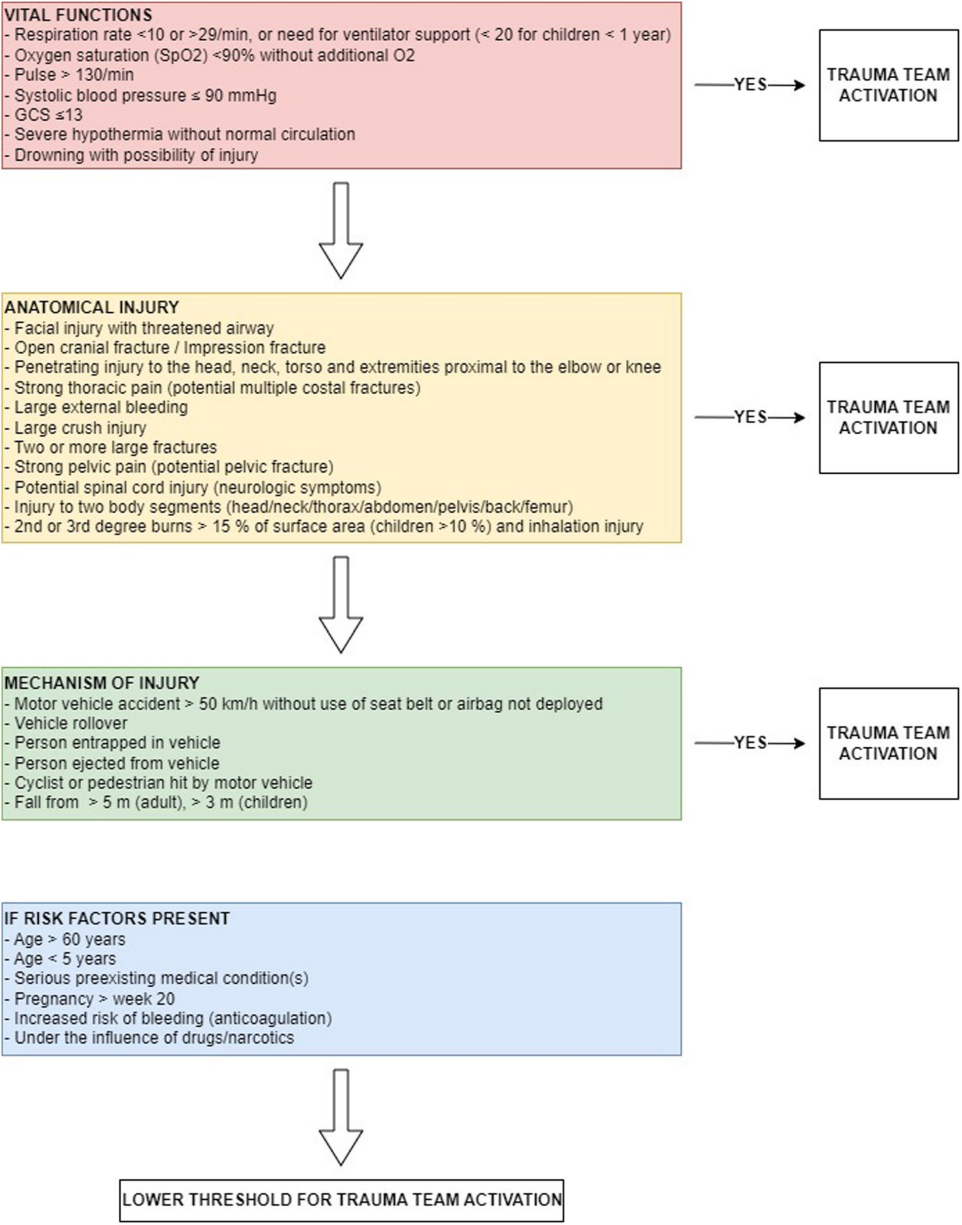
Overview of National Trauma Criteria (NTrC). The illustration gives an overview of the national trauma criteria (NTrC) in use at the time of study. The flowchart provide a step-step decision making chart based on the importance of groups of criteria where vital functions are seen as the most sensitive criteria to detect severe injury

Norway’s health services and trauma system are organized into four regions. Each region comprises one regional trauma center (TC) and multiple acute care hospitals (ACH) [12]. At the time of study, 38 hospitals admitted trauma patients, of which four were TCs and 34 ACHs. The two-level hospital categorization has been derived from the ACS-COT accreditation, where TCs are comparable to Level 1 and 2 TCs and most ACHs are similar to Level 3 hospitals [12, 16] A detailed overall description of the Norwegian trauma system has been published previously [16]. In 2015, common NTrC were established, recommending the use of 31 specific criteria divided into the following four groups: physiologic variables (n = 7), anatomical injury (n = 11), mechanism of injury (MOI) (n = 7) and risk factors (n = 6) [12] (Fig. 1). According to the Norwegian Trauma Systems Framework, a TTA prior to patient arrival is aimed for, but TTA can also be performed after hospital arrival if the patient fulfils TTA criteria [12].

Detailed data of number of annual TTA, overtriage and undertriage were not accessible for the time period investigated (2022) and was not requested as part of the survey. However, compiled and categorized data for the year 2023 [17] together with questionnaire data is presented in Additional File 1. Although data presented were from two different time periods, longitudinal data presented in the annual trauma registry report shown minimal timely variations [17]. A total of 8657 TTAs were performed in Norwegian hospitals in 2022.

### Data collection

The study collected data using a secure digital questionnaire service *Nettskjema* (https://nettskjema.no/), a solution developed and hosted by the University of Oslo [18]. Local hospital trauma coordinators were invited to provide information specific to their hospital. A short explanation of the aims of the study was provided digitally prior to consenting and filling out the survey. Data collection took place between April and September in 2022, with three reminders (i.e. follow-up emails and/or telephone calls) to non-responders or in the need to clarify answers. Thirty-eight hospitals were eligible for inclusion and the investigators were able to collect additional responses through telephone communication as well as complement several incomplete responses. The survey was written in Norwegian and contained a semi-structured questionnaire consisting of eleven questions and requested files received from the respondents (Additional File 2). The survey consisted of questions relating to the following: (1) adherence to national trauma triage criteria, (2) tiered trauma team activation, (3) trauma team activation (TTA) process, and (4) documentation process of used trauma triage criteria.

### Data analysis

The collected data was systematized and analyzed using Excel (© Microsoft 2023, Redmond, Washington, USA) and compared to the current NTrC to uncover any potential discrepancies between the respondents’ answers and the NTrC [12]. Descriptive statistics are presented as frequencies, percentages and averages where appropriate.

The investigators analyzed adherence to NTrC by asking hospitals if the use of trauma triage criteria for TTA in their respective hospitals corresponded with the NTrC. If they verified, the participants were required to provide written documentation outlining the used triage criteria. Each hospital-provided criterion was compared to the NTrC. The investigators regarded a hospital adhering fully to a criterion only if the hospital-provided criteria corresponded, and the words were identical to the wording the NTrC. Supplementary questions were included to provide an overview of the trauma triage decision-making process within the responding hospitals.

### Ethics

The study was reviewed and approved by the Regional Ethics Committee for medical and health research (REC; reference number 453816) and the Norwegian Centre for research data (NSD; reference number 836580). After being provided with written information of the study, participants were required to digitally consent prior to answering the study questions.

## Results

### Response rate

Thirty-five out of 38 acute care trauma hospitals responded, yielding a 92% response rate.

### Adherence to national trauma triage criteria

**Fig. 2:**
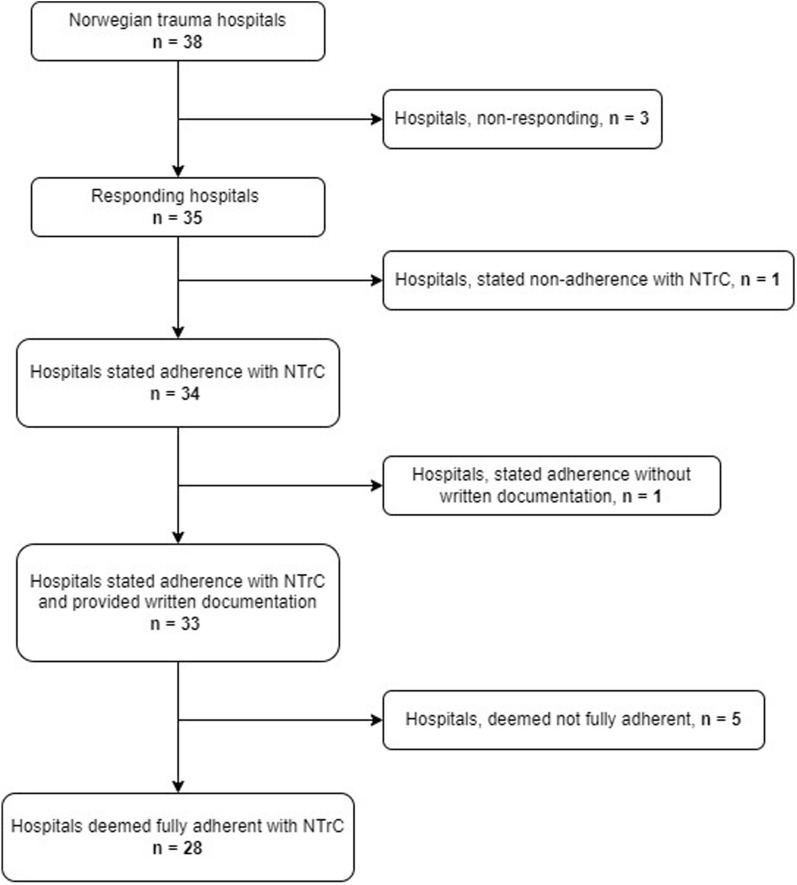
Inclusion flowchart. The illustration gives an overview of the inclusion process. NTrC: national trauma criteria

Thirty-four of the 35 responding hospitals stated that they adhered to the NTrC of which one hospital did not provide written documentation. Thirty-three provided written, printed or digital copies of their criteria for TTA. Out of the thirty-three hospitals with documented criteria, twenty-eight (80%) hospitals adhered fully to the NTrC (Fig. 2). We observed no relations between the level of adherence and corresponding overtriage/undertriage rates among hospitals (Additional File 1).

Of the five respondents deemed not adhering to the NTrC, one hospital did not specify the degree of burn injury, only the affected surface area. One hospital did not use the criteria “drowning with possibility of injury”. Two hospitals used 28 instead of 29 as the upper respiratory rate threshold. One hospital was deemed not adherent since a Glasgow Coma Scale (GCS) threshold criterion of 12 was used instead of 13 as the lower limit for GCS. In addition the same hospital used 28 instead of 29 as the higher limit for the respiratory rate.

### Tiered trauma team activation

Three of the 35 hospitals (8.6%) (one TC and two ACHs) used a tiered partial TTA approach with different sized teams. Thirty-two (91.4%) hospitals only used one-size defined trauma team. In addition, four of the 35 hospitals (11.4%) (four ACHs) used specialized teams in addition to the trauma team to meet the needs of defined patient groups at risk, such as geriatric patients and traumatic brain injury.

### Trauma team activation process

Twenty-one (60%) of the responding hospitals had written guidelines on who could request TTA. In 33 hospitals several professions (≥ 2) could request the trauma team and in two hospitals only the leading on-call emergency department nurse could request TTA. In addition to in-hospital TTA, eighteen hospitals (51.4%) allowed for pre-hospital (i.e. paramedics and air ambulance physician) TTA. Among pre-hospital personnel, HEMS crew and anesthesiologist-staffed rapid response car could request TTA in 15 hospitals (43%). Table 1 shows the in-hospital professions who could perform TTA.

**Table 1:** Overview of in-hospital professions who could request trauma team activation

Profession	Number of (N = 35)
Trauma team leader	30 (85.7%)
Emergency department nurse	29 (82.9%)
Emergency department trauma coordinator	20 (57.1%)
On call—surgeon	20 (57.1%)
On call—anesthesiologist	17 (48.9%)
Other	10 (28.6%)

### Documentation process

Seventeen (48.6%) hospitals informed clinicians on which NTrC was fulfilled in each trauma case (using digital tools), while 18 (51.4%) did not. In eight (22.9%) hospitals the used TTA criteria were documented electronically, 15 (42.9%) hospitals used paper-based documentation and 12 (34.2%) hospitals did not document the criteria that led to TTA in each case. Two (5.7%) of the respondents documented electronically who requested TTA while eight (22.9%) documented this on paper. Twenty-five (71.4%) of the respondent’s stated that they did not document who requested TTA in each case.

## Discussion

This study, with a high response rate, show that the majority of Norwegian trauma hospitals adhere to the national criteria for activation of the trauma team implemented in 2015. However, the study also reveals that there are considerable differences in how trauma teams are activated, who activates them and how the use of trauma triage criteria are documented.

### Adherence to national criteria

In 2010, Larsen et al. investigated potential differences in TTA criteria among Norwegian hospitals. They found large variations among the 49 included hospitals, where no single criteria were common to all hospitals, with a wide variation of physiological threshold values and 156 different criteria used [3]. Since then, the establishment of a national trauma systems framework, which was last revised in 2023 [12], a Norwegian trauma registry (2015) [19] and the Norwegian National Advisory Unit on Trauma [20] have contributed to system improvements for the care of the severely injured. The implementation of these formal bodies and their related continuous quality assessments are likely to have provided a more common understanding of the need for standardization. Our findings show a notable systemic improvement compared to previous findings. When comparing rates of overtriage and undertriage among hospitals, we observed no relations between these and the level of adherence (Additional File 1). The establishment of uniform guidelines, a national trauma system framework and continuous quality assessment might provide a reasonable explanation to the strong adherence to NTrC. A study from The Netherlands in 2015 surprisingly found that only 38% of the hospitals adhered to a pre-defined TTA protocol, despite the existence of national guidelines [21]. In 2017, a new TTA algorithm based on a modified two-tier ACS-COT trauma triage criteria; The Swedish National Trauma Triage Criteria (SNTTC), was introduced in Sweden [22]. The safety and efficacy of SNTTC were investigated in a study published in 2019, but did not investigate the nationwide adherence of this algorithm [23]. In 2018, a Danish nationwide study found that, although all hospitals had formalized TTA criteria, the criteria were diverse between the hospitals [24]. The majority (68%) of hospitals used a point-scoring system, whereas 23% used a more traditional stepwise approach using the criteria outlined by ACS-COT and the remaining 4.5% used a combination of the two systems [24]. The authors recommended the establishment of a national consensus based on uniform and evidence-based criteria [24]. A recent Canadian study showed great variability among TTA in hospitals, with no overarching structure providing national guidelines [25]. In 2018, van Rein and colleagues underlined the need for improvement of triage protocols and compliance rate in a systematic review [26]. Findings from their included studies showed a variation in adherence rates from 21 to 93% for triage protocols, and 41% to 94% for specific criteria categories [26]. The authors’ focus was on the adherence of triage protocols on an EMS provider level and not on the implementation on a system level. Therefore, when evaluating adherence to protocols it is important to distinguish between studies that evaluate the adherence of TTA protocols on system versus provider level.

In 2021, a new revised national US guideline for the field triage of injured patients based on the most updated science was published [27]. Previous guidelines were slow to be implemented among providers despite the use of evidence-based data and consensus processes [28, 29]. The authors described that only 17% of the states had implemented the revised guidelines, while another 61% used an older or other protocol [28]. Therefore, the authors underlined the importance of “creating new ways of disseminating, implementing, and monitoring adherence to realizing the true potential of the guideline” [27]. Based on our results and experiences from other countries, we argue that a national all-encompassing organizational structure supplying national guidelines and providing consistent verification and review, will result in a more unified common approach to the severely injured trauma patient. This also underlines the common challenge and need for uniform protocols across countries, regions and systems.

### Tiered Trauma Teams and special teams

In the Norwegian system, no formal description of a multilevel-tiered trauma response is described [12]. The 2014-guidelines from ACS-COT recommended the use of tiered TTA, which again was underlined in the 2021-revised guidelines [6, 27]. A study published in 2012 by Rehn et al. described a beneficial effect of a tiered TTA by reducing the undertriage from 28.4 to 19.1% [8]. In 2022, a new study within the same hospital found increased undertriage after re-introduction of a single tiered response and the authors concluded that a two-tiered TTA provided better patient care [30]. The NTR Annual report from 2020 showed that undertriage in trauma is a common event in Norway, and any intervention to reduce these rates should be evaluated [31]. In our study, we found that the majority of Norwegian hospitals employ a one-tiered TTA system, while only three hospitals use a tiered approach. The development of these teams should be observed in combination with local and historical traditions, and might reflect specific needs, local administrative adjustments or adherence to protocols recommended in the literature. However, these results are comparable to previous studies, showing national variations in mature trauma systems [21, 25].

### Decision making

In our study, we found that that there is a good institutional adherence to NTrC. However, knowledge on the individual clinician’s adherence is also important to evaluate on efficiency and precision of specific TTA criteria [32]. Several investigations have looked into the precision of TTA criteria [27], though there is sparse knowledge on the actual TTA process and who actually requests trauma team activation. Our study found that the organizational structure allows for different practices among hospitals and even within hospitals, as different professions are involved in the TTA decision. In a study by Gutacker et al., they investigated how interventions to reduce unwanted practice variation affected individual clinicians and hospitals [33]. They concluded that practice variation were greater among clinicians compared to their attributable hospitals, even when taken the amount of case-mix adjusted patient variation into account [33]. In our study, most hospitals allowed for several professions to request for TTA. This enables practice variation among hospitals on TTA, which in combination with individual interpretation of TTA criteria may lead to different practice patterns among hospitals. When comparing quality indicators among hospitals which is likely to be affected by these factors, one must consider which interventions should be introduced to reduce clinical practice variation.

### Documentation

Continuous assessment of clinical practices requires valid documentation. We found considerable variations on how the use of different TTA criteria were documented. In addition, 71% of the respondents did not document who requested TTA in each case. Even though there is scarce knowledge on these subjects in the existing literature, we suspect the lack of documentation is prevalent in other systems as well. Although there is a high adherence to NTrC, several vitals elements regarding decision making process and documentation needs to be addressed. Several studies have been performed into the precision (i.e. sensitivity and specificity) of specific trauma criteria [27], though their validity and ability to assess quality of care can be questioned if their use is not consistent among institutions. Wennberg stated “there is variation in the utilization of health services that cannot be explained by variation in patient illness or patient preferences” [34]. We argue, that awareness of potential differences in the decision-making process of TTA is as important as the specific criteria themselves. TTA is a dynamic process as information pertaining to the event itself will increase as time passes. Uncertainties during this process imply that not only the TTA criteria, but also the process, accessible information and inter-individual differences may influence the results of TTA. The study results uncover differences in the decision-making process of TTA and there are currently no national guidelines on who can request TTA, or the dynamics of people involved in the individual trauma care.

### Strengths and limitations

The first limitation of the study was the design where surveys investigating a practice may differ from actual integration in clinical practice for a variety of reasons. Surveys are based on self-reported data, where respondents may not have knowledge on all aspects of the subject to be investigated and may be prone to recall-bias. However, a cross-sectional design was easy to perform and allowed for sampling of knowledge from respondents involved in the everyday care of these patients. Secondly, the survey may not have been designed to capture all facets of the integration process, including elements that are not readily apparent to the participants or are not part of the survey's focus (e.g., patient education). A strength of the study is the high response rate, allowing us to include the majority of Norwegian hospitals admitting trauma patients, providing us with a good overview of national implementation. Another strength is that the respondents were dedicated health personnel who worked as either trauma registrars or trauma coordinators, and for this reason were well acquainted with the criteria and the hospital's routines.

## Conclusion

There is good adherence to the national criteria for activation of the trauma team among Norwegian trauma hospitals after implementation of national guidelines. Individual hospitals argue the use of certain local criteria and trauma team activation decision-making processes to increase their precision in specific patient populations and demographics. Further steps should be taken to reduce the variation in TTA decision-making processes among hospitals and improve documentation quality.

## Supplementary Information

Supplementary Material 1.

Supplementary Material 2.

## Data Availability

Data is available upon reasonable request to the corresponding author.

## Abbreviations

ACH: Acute care hospital
ACS-COT: American College of Surgeons—Committee on Trauma
EMS: Emergency medical services
GCS: Glasgow Coma Scale
ISS: Injury Severity Score
MOI: Mechanism of injury
NTrC: Norwegian national criteria for activation of the trauma team
NTR: National Trauma Registry
REC: Regional Ethics Committee
SNTTC: Swedish National Trauma Triage Criteria
STROBE: Strengthening the Reporting of Observational Studies in Epidemiology
TC: Trauma Center
TTA: Trauma Team Activation
