# Supplementary material for: Adherence to national trauma triage criteria in Norway: a cross-sectional study
Source: Scand J Trauma Resusc Emerg Med. 2024 Dec 18;32:133. doi: 10.1186/s13049-024-01306-x (PMC11656868; doi:10.1186/s13049-024-01306-x)
Supplement: Supplementary file 2 — Supplementary Material 2. [file 13049_2024_1306_MOESM2_ESM.pdf]

Additional File 2: Overview of survey questions

Question 1

What is your role in trauma care at the responding hospital?

o Trauma registrar

o Trauma coordinator

o Other

If other, specify (free text field)

Question 2

Do you follow the national field trauma criteria for trauma team activation? (one answer possible)

o Yes

o No

Question 3

Does your hospital have a written document procedure displaying trauma team activation criteria? (one answer possible)

o Yes

o No

If yes, attach a list of the criteria in use for trauma team activation. (option to upload documents)

Question 4

Do you have tiered trauma team activation and/or specified teams to treat defined patient groups?

o No – only one team

o Yes – tiered activation (small and large team)

o Yes – specified team

If yes – specified team, specify in free text (free text field)

Attach an overview of the types of teams employed and the members of these teams. (option to upload documents)

Question 5

Who in your hospital can request trauma team activation? (multiple selections possible)

o Trauma team leader

o Emergency department nurse

o Emergency department trauma coordinator

o On call surgeon

o On call anaesthesiologist

o Other (specify in free text field)

Question 6

Are there other employees in the hospital that can request trauma team activation? Specifically, those who can request the trauma team, not those who suggest or recommend activations but without the authority. (One answer possible)

o Yes

o No

If yes, select who (multiple selections possible):

o Emergency medical coordination centre

o Helicopter emergency medical services

o Emergency medical services

o Other (specify in free text field)

Question 7

Are the specific trauma team activation criteria in each trauma case documented? (one answer possible)

o Yes – electronically

o Yes – on paper

o No

Question 8

Is it documented in each trauma case who requests the trauma team? (one answer possible)

o Yes – electronically

o Yes – on paper

o No

Question 9

Can a decision to activate a trauma team be challenged in your hospital? (one answer possible)

o Yes

o No

If yes, who can challenge the activation? Specify in free text (free text field)

Question 10

Do you have standard operating procedure on who can request the trauma team in your hospital? (one answer possible)

o Yes

o No

If yes, who can activate the trauma team? Specify in free text and attach your protocol (free text field and option to upload documents)

Question 11

Do you have digital information available to inform involved trauma care personnel on which trauma criterion have been used in a trauma call? (one answer possible)

o Yes

o No

If yes, which personnel? (multiple options possible)

o Emergency medical services

o In-hospital staff

o Emergency medical coordination centre

Specify in free text (free text field)
